# Physicochemical Properties and Surfaces Morphologies Evaluation of MTA FillApex and AH Plus

**DOI:** 10.1155/2014/589732

**Published:** 2014-05-04

**Authors:** Álvaro Henrique Borges, Maura Cristiane Gonçales Orçati Dorileo, Ricardo Dalla Villa, Alexandre Meireles Borba, Tereza Aparecida Delle Vedove Semenoff, Orlando Aguirre Guedes, Cyntia Rodrigues Araújo Estrela, Matheus Coelho Bandeca

**Affiliations:** ^1^Faculty of Dentistry, University of Cuiabá, Avenida Manoel José de Arruda 3.100, Jardim Europa, 78065-900 Cuiabá, MT, Brazil; ^2^Master Program in Chemistry, Mato Grosso Federal University, Avenida Fernando Corrêa da Costa 2367, Boa Esperança, 78060-900 Cuiabá, MT, Brazil; ^3^Master Program in Dentistry, UNICEUMA, Rua Josué Montello 01, Renascença, 65075-120 São Luís, MA, Brazil

## Abstract

The solubility, pH, electrical conductivity, and radiopacity of AH Plus and MTA FillApex were evaluated. In addition, the surfaces morphologies of the sealers were analyzed by using scanning electron microscopy. For pH test, the samples were immersed in distilled water at different periods of time. The same solution was used for electrical conductivity measurement. The solubility and radiopacity were evaluated according to ANSI/ADA. Statistical analyses were carried out at 5% level of significance. MTA FillApex presented higher mean value for solubility and electrical conductivity. No significant difference was observed in the mean values for pH reading. AH Plus presented higher radiopacity mean values. MTA FillApex presented an external surface with porosities and a wide range of sizes. In conclusion, the materials fulfill the ANSI/ADA requirements when considering the radiopacity and solubility. AH Plus revealed a compact and homogeneous surface with more regular aspects and equal particle sizes.

## 1. Introduction

The success of endodontic therapy is related to the removal of dentine tissue to promote cleaning and disinfection, as well as to prepare root canal system to receive the filling material [1]. Complete root filling is achieved by the three-dimensional obturation of the root canal system with the association of a solid filling material to the endodontic sealer [2]. Thus, it is imperative to eliminate the empty spaces inside the tooth, which can harbor the presence of tissue fluid and microorganisms. In this context, it allows tissue repair, because the periapical tissues are able to rest from the previous irritation; it favors osteogenesis and cementogenesis, followed by the reorganization of the periodontal ligament and reintegration of the lamina dura [3]. The sealers commercially available are classified according to chemical components: zinc-oxide-eugenol sealers, sealers containing calcium hydroxide, and resin-based, glass-ionomer-based, silicone-based, and bioceramic sealers [2–4].

AH Plus (Dentsply DeTrey, Konstanz, Germany) is a hydrophobic epoxy resin-based sealer that has been used as the gold standard for comparisons with other endodontic sealers [4]. Considering the stability, this material presents smaller dimensional changes. On the other hand, its sealing ability is compromised in function of the difficulty to bond to gutta-percha [5] and in the presence of moisture, the material does not efficiently adhere to canal walls [6].

MTA FillApex (Angelus, Londrina, PR, Brazil), a MTA-based endodontic sealer, was introduced recently in the market. According to the manufacturer, after the mixing, the composition of the material is essentially MTA, salicylate resin, natural resin, bismuth oxide, and silica nanoparticles. Several properties of MTA FillApex such as biocompatibility [7, 8], bioactivity [9], cytotoxicity [10], solubility [11–13], antibacterial effect [14], and sealing ability [15] have been investigated, but more information about the material is required. In this way, it is imperative to know more about the physicochemical properties of MTA FillApex and its possible use in clinical practice.

The purpose of this in vitro study was to assess the solubility, hydrogenionic potential, electrical conductivity, and radiopacity of 1 epoxy-amine resin sealer (AH Plus) and 1 MTA-based sealer (MTA FillApex), according to ANSI/ADA standards. In addition, the surfaces morphologies of the sealers were analyzed by using scanning electron microscopy.

## 2. Materials and Methods

The materials evaluated in the present study and the chemical compositions, according to the manufactures, are described in Table 1.

The solubility, pH, electrical conductivity, and radiopacity were determined in accordance with methods recommended by the ANSI/ADA specification number 57 [16] for endodontic sealing materials and as suggested by Carvalho-Junior et al. in 2007 [17]. The cements were manipulated according to the manufacturer's instructions.

### 2.1. Solubility Test

Five samples (1.5 mm thickness and 7.75 mm inner diameter) were used for each material. The tested material was prepared and inserted into the mold. In sequence, a 0.5 mm diameter waterproof nylon was inserted in the softened cement. After three times the setting time, the sample was removed from the mold and weighed on a precision scale of 0.0001 g (Ohaus Corporation, Parsippany, New Jersey, USA). The sample suspended by the nylon was placed in a wide-mouthed plastic recipient containing 7.5 mL of distilled water and was maintained hermetically closed in an incubator at a constant temperature of 37 ± 2°C for 24 h. After this time, the sample was removed and the excess water was removed with absorbent paper. The sample was maintained in dehumidifier for 24 h, after which it was weighed a second time. The material's solubility was considered as the percentage of lost mass compared to the initial mass. Five repetitions were considered for each material.

### 2.2. pH Analysis

Five samples (1.5 mm thickness and 7.75 mm inner diameter) were used for each material. Each cylinder was sealed in a flask containing 7.5 mL of distilled water. Distilled water pH measurements were taken with a pH meter (Corning Inc, Corning, New York, USA) at 1, 3, 5, 15, and 30 min, 1, 2, 3, 4, 6, 9, 12, 24, 48, 72 h, 4, 6, 7, 15, and 30 days after spatulation. During the experiment, pH was analyzed for each sample in the same plastic recipient without liquid substitution. It was measured five times for each material. Mean values and standard deviations were recorded for all measurements.

### 2.3. Electrical Conductivity Analysis

After the pH analysis, the sample was retained in the plastic recipient and the electrical conductivity of the solution was measured. All 5 samples of each material were analyzed with a condutivimeter (Marconi Equip. Ltda, Piracicaba, São Paulo, Brazil). The device was calibrated according to a calibration curve obtained from a solution of 1.412 *μ*S/cm^−1^.

### 2.4. Radiopacity Test

Five acrylic plates (2.2 cm × 4.5 cm × 1 mm) with 6 holes measuring 1 mm in depth and 5 mm of internal diameter were fulfilled with the tested cements. For the radiographic exposure, each acrylic plate containing the cements was positioned together with another acrylic plate (1.3 cm × 4.5 cm × 1 mm), which contained a graduated aluminum stepwedge varying from 1 to 10 mm in thickness, and uniform steps of 1 mm each. The set of plates corresponds exactly to the sensor size from Digora system (Soredex Orion Corporation, Nilsiänkatu, Helsinki, Finland), used for data collection. A 70 kVp and 8 mA radiograph machine Spectro 70X (Dabi Atlante Ind. Méd. Odontol. Ltda, Ribeirão Preto, São Paulo, Brazil) was used. The focus-object distance was 30 cm and exposure time was at 0.2 s. The sensor, after being exposed, was inserted into the laser optical reader of Digora for Windows 5.1 software. The same phosphor plate was used for all exposures. The system performed a radiographic density reading over images of each cement revealed on screen, and also a reading of steps on an aluminum stepwedge, resulting in a numeric value for each reading. After evaluating the 5-acrylic set of plates, 5 measurements for each type of cement and for each step of the aluminum scale were obtained. Mean values were taken by a single evaluator previously trained and blinded with regard to the different groups.

### 2.5. Scanning Electron Microscopy Examination (SEM)

For SEM examination, cylindrical Teflon moulds (3 × 4 mm) were filled with freshly mixed sealers. The moulds were supported by a glass plate covered with a cellophane sheet and placed in a chamber (37°C, 95% relative humidity) for a period corresponding to three times the setting time. After that, the samples were sprinkled on carbon double-sided tape over a metallic stub, critical-point dried, and sputter-coated with gold palladium (Bal-Tec AG, Balzers, Liechtenstein, Germany) at 20 mA. The surfaces morphologies of the samples were qualitatively analysed under a field emission SEM (JSM-6610; Jeol Ltd., Akishima, Tokyo, Japan) at an accelerating voltage of 8–10 kV, a working distance of 15 mm, and at ×50 and ×500 magnifications.

### 2.6. Statistical Analysis

For each test, the data were statistically analyzed by one-way analysis of variance and the Tukey's test at 5% level of significance, with Kolmogorov-Smirnov and Levene tests (normality and variances homogeinity). The tests were performed with the IBM SPSS for Windows statistical software version 21 (SPSS Inc., Chicago, Illinois, USA).

## 3. Results

Table 2 presents the mean values and standard deviations of the physiochemical properties of the tested materials.

### 3.1. Solubility

MTA FillApex presented higher mean value for solubility (Table 2) while AH Plus presented lower mean value, with significant differences between them (*P* < 0.05).

### 3.2. pH

**Figure 1:**
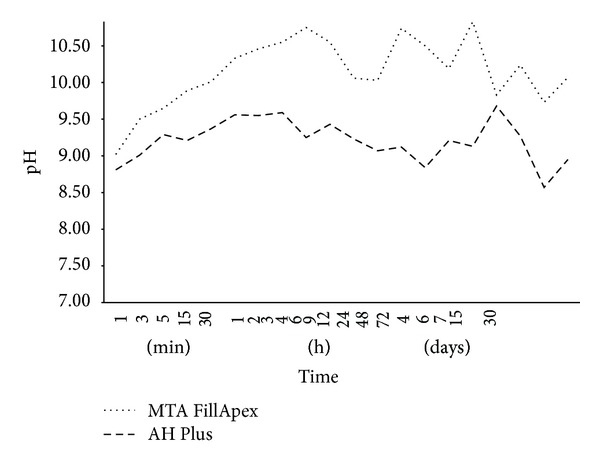
Hidrogenic potential changes of the tested materials according to different periods of time.

No significant difference was observed in the mean values for pH reading of each tested material (*P* > 0.05) (Table 2). The change in pH as a function of time is shown in Figure 1. The pH values for the cements ranged from 8.84 to 11.70. At 1 min immersion, significant differences were observed from those of other time periods (*P* < 0.05).

### 3.3. Electrical Conductivity

**Figure 2:**
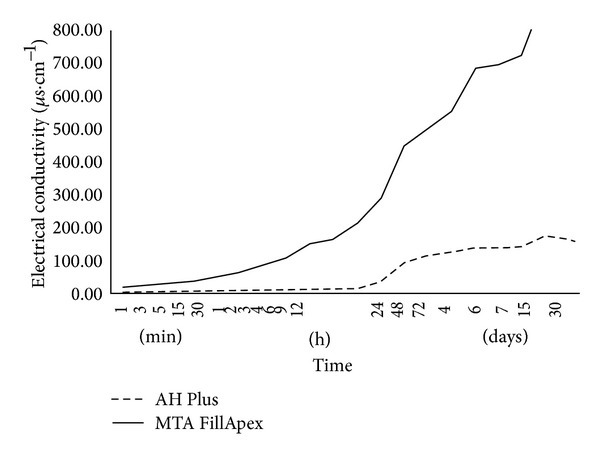
Electrical conductivity (*μ*S/cm^−1^) evaluation according to different periods of time.

The results indicated that the conductivity of the materials was statistically different (*P* < 0.05) (Table 2). At all periods of time, differences were observed between samples (*P* > 0.05) (Figure 2).

### 3.4. Radiopacity

AH Plus presented higher radiopacity mean values and statistical analysis demonstrated difference between the tested materials (*P* < 0.05). Both cements overcame 3 steps from the aluminum stepwedge, which is the minimum recommended by the ANSI/ADA [15] (Table 2).

### 3.5. SEM Evaluation

**Figure 3:**
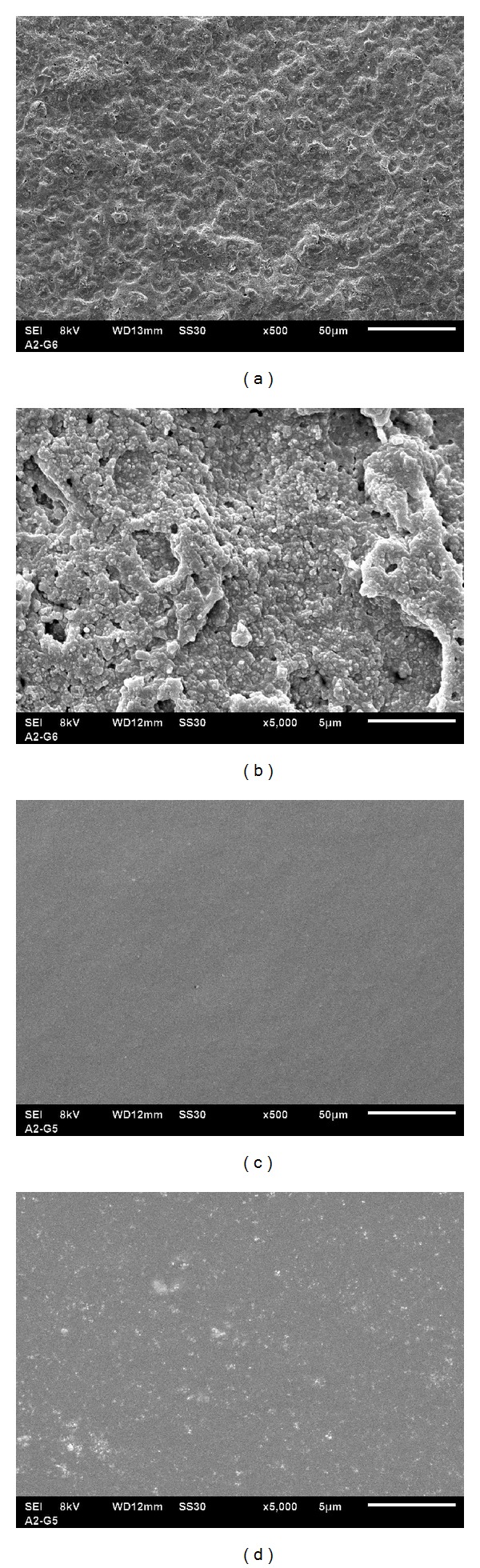
Photomicrographs of surfaces morphologies of the sealers analyzed by using scanning electron microscopy. MTA FillApex magnification of 500x (a) and 5000x (b). AH Plus magnification of 500x (c) and 5000x (d).

Selected photomicrographs obtained from the samples about their morphological appearance are presented in Figure 3. MTA FillApex had an external surface that appeared to be mostly homogeneous rough surface with porosities and a wide range of sizes (Figures 3(a) and 3(b)). AH Plus revealed a compact and homogeneous surface with more regular aspects and equal particle sizes (Figures 3(c) and 3(d)).

## 4. Discussion

Numerous researches have been proposed to compare the biological and physicochemical properties of MTA to other sealers. Classically, AH Plus is considered a gold standard reference when considering the physicochemical properties of a sealer for root canal filling. For this purpose, recently MTA FillApex, a calcium silicate cement, is being investigated in consequence of its good properties as an endodontic sealer combined with the biological properties of MTA [18]. In the present study, the solubility, pH, electrical conductivity and radiopacity of AH Plus were analyzed and compared to MTA FillApex.

In general, sealing materials should be low soluble in contact with tissue fluid. In cases of materials that present high solubility, chemical compounds can be released and then irritate periapical tissues. The possibility to form gaps between root canals and filling mass can be also considered, favorable to increase bacterial leakage [19]. The tests were determined according to methods prescribed by ANSI/ADA [16] for endodontic sealing materials and as suggested by Carvalho-Junior et al. [17], allowing the reduction of 80% in volume of material for conducting tests, with no interference in results. The findings evidenced that the solubility of AH Plus was statistically lower than that of MTA FillApex even though both materials fulfill the ANSI/ADA [16], according to which a root canal sealer should not present solubility higher than 3%. The results observed in this study are in agreement with the literature but it is important to consider that in vitro solubility studies are tightly different from clinical situations, and then higher values may be found [19–22].

Both materials evaluated in this study promoted an alkaline pH when immersed in distilled water, with values ranging from 7.30 to 11.35, which remained high until the end of the experiment. The pH of MTA FillApex was higher over the period of the test. MTA-based cements are rich in calcium ions [20], which are converted to calcium hydroxide upon contact with the water, and dissociate into calcium and hydroxyl ions, increasing pH of the solution [21]. Thus, the variation in the concentration of calcium hydroxide leads to different pH values [23]. A high pH activates alkaline phosphatase, an enzyme strictly involved in the mineralization process [20, 22], and also neutralizes the acids secreted by osteoclasts, avoiding the destruction of mineralized tissue [18].

Electrical conductivity is related to the quantity of ions released to medium and the facility that each material has to conduct its own electric charge [24]. It is directly proportional to material solubility and the components that were the most soluble in water were the first to release ions into the solution [24, 25]. The results of the present study indicated that the concentration of ions in solution increased as the solubility of the sample increased, which led to higher conductivity values during the period of test. The highest values of MTA FillApex electrical conductivity is probably related to its highest solubility [19–22]. Considering the time, MTA FillApex presented values significantly higher over the period of the tests. In the present study, the solution was not removed or exchanged once the samples were immersed.

To be easily distinguishable from dentin and gutta-percha on radiographs, the root filling material should present radiopacity equivalent to step 3 of the aluminium stepwedge, which is correspondent to 3 mmAl (ANSI/ADA) [16]. According to the present results, AH Plus was the most radiopaque material which is in accordance to the literature [26, 27]. These findings are probably by the presence of different radiopacifying agents in each material. AH Plus has calcium tungstate and zirconium oxide in its composition [28] and this association produces superior radiopacity [29]. The bismuth oxide in the MTA FillApex's composition is responsible for the less radiopacity of the material [30]. MTA-based cements contain approximately 13.63 to 16.9 wt.% of bismuth. Both MTA FillApex and AH Plus were found to be in agreement with ANSI/ADA recommendations referring to radiopacity [16].

SEM is a powerful technique applied in microimaging to explore the surface of a solid sample which is scanned in a raster pattern with a beam of energetic electrons. The surface structure morphology, by the particle size or granulation, is an important characteristic feature of the physical properties [22, 23]. The differences in the particle size of the materials tested are of great importance for the mechanical characteristics. With a similar particle size a higher mechanical strength is designed by a reduced spreading in grit size [31], which it could be observed more in AH Plus than in MTA FillApex. The physical structure and surface characteristics of the material associated to its cytotoxicity are probably related to biocompatibility [32]. The results presented in this study are very likely to explain why AH Plus is considered a good biocompatible material [33] and also enforce the morphological behavior of human periodontal ligament fibroblasts on MTA-based cements [31].

## 5. Conclusion

Based on the results of this study, it seems that all tested materials presented solubility in accordance to ANSI/ADA. No difference about the pH solution was observed among the cements and it maintained alkaline over the period of test. MTA FillApex presented higher electrical conductivity. Both MTA FillApex and AH Plus met the ANSI/ADA recommendations referring to radiopacity. The SEM images showed that the morphologies of MTA FillApex are composed of particles with a wide range of size, whereas AH Plus showed a uniform and smaller particle size.

## Figures and Tables

**Table 1:** Composition of the materials and their manufacturers.

Material	Materials composition (MSDS data)	Manufacture
AH Plus	Paste A: bisphenol A epoxy resin, bisphenol F epoxy resin, calcium tungstate, zirconium oxide, aerosol, and iron oxide Paste B: dibenzyldiamine, adamantane amine, tricyclodecane diamine, calcium tungstate, zirconium oxide, aerosol, and silicon oil	Dentsply DeTrey GmbH, Konstanz, Germany
MTA FillApex	After the mixture: salicylate resin, natural resin, diluting resin, bismuth oxide, nanoparticulated silica, MTA, and pigments	Angelus Soluções Odontológicas, Londrina, Paraná, Brazil

**Table 2:** Physicochemical properties of the tested materials (mean ± standard deviation).

Test	Tested materials	
	AH Plus	MTA FillApex
Solubility (%)	0,56 ± 0,48^a^	2,88 ± 0,48^b^
pH	9,08 ± 0,66^a^	9,97 ± 0,90^a^
Electrical conductivity (µS/cm^−1^)	62,83 ± 65,88^a^	273,16 ± 251,65^b^
Radiopacity (mm Al)	193,80 ± 7,82^a^	172,00 ± 7,42^b^

*Different superscript letters represent statistically significant difference (*P* < 0.05).
